# Changes in Serum IgG Glycosylation Patterns for Abdominal Aortic Aneurysm Patients

**DOI:** 10.3390/jcdd9090291

**Published:** 2022-09-01

**Authors:** Siting Li, Jingjing Meng, Yanze Lv, Qian Wang, Xinping Tian, Mengtao Li, Xiaofeng Zeng, Chaojun Hu, Yuehong Zheng

**Affiliations:** 1 Department of Vascular Surgery, Peking Union Medical College Hospital, Chinese Academy of Medical Sciences and Peking Union Medical College, Beijing 100010, China; 2 Department of State Key Laboratory of Complex Severe and Rare Diseases, Peking Union Medical College Hospital, Chinese Academy of Medical Science and Peking Union Medical College, Beijing 100010, China; 3 Department of Rheumatology, Peking Union Medical College Hospital, Peking Union Medical College and Chinese Academy of Medical Sciences, National Clinical Research Center for Dermatologic and Immunologic Diseases (NCRC-DID), Key Laboratory of Rheumatology & Clinical Immunology, Ministry of Education, Beijing 100730, China; 4 Department of Clinical Laboratory, Fifth Affiliated Hospital of Zhengzhou University, Zhengzhou 450052, China

**Keywords:** glycosylation, abdominal aortic aneurysm, lectin microarray, biomarker

## Abstract

**Background:** B cells and autoantibodies play an important role in the pathogenesis of abdominal aortic aneurysm (AAA). IgG glycosylations are highly valued as potential disease biomarkers and therapeutic targets. **Methods:** Lectin microarray was applied to analyze the expression profile of serum IgG glycosylation in 75 patients with AAA, 68 autoimmune disease controls, and 100 healthy controls. Lectin blots were performed to validate the differences. The clinical relevance of lectins binding from the microarray results was explored in AAA patients. **Results:** Significantly lower binding level of SBA (preferred GalNAc) was observed for the AAA group compared with DCs (*p* < 0.001) and HCs (*p* = 0.049). A significantly lower binding level of ConA (preferred mannose) was observed in patients with aneurysm diameter >5 cm. Significantly higher binding of CSA (preferred GalNAc) was present for dyslipidemia patients, whereas a lower binding level of AAL (preferred fucose) was observed for hypertensive patients. Patients with diabetes had lower binding levels of IRA (preferred GalNAc) and HPA (preferred GalNAc) compared with those not with DM. PTL-L (R = 0.36, *p* = 0.0015, preferred GalNAc) was positively associated with aneurysm diameters, whereas DSL (R = 0.28, *p* = 0.014, preferred (GlcNAc)2-4) was positively associated with patients’ age. Symptomatic patients had a lower binding level of ConA (*p* = 0.032), and patients with coronary heart disease had higher binding levels of STL (*p* = 0.0029, preferred GlcNAc). Patients with ILT bound less with black bean crude (*p* = 0.04, preferred GalNAc). **Conclusions:** AAA was associated with a decreased IgG binding level of SBA (recognizing glycan GalNAc). Symptomatic patients with aneurysm <5 cm had a higher binding level of ConA (preferred mannose). Coronary heart disease and elder age were associated with increased IgG bisecting GlcNAc. IgG O-glycosylation (GalNAc) may play an important role in AAA pathogenesis and progression.

## 1. Introduction

Abdominal aortic aneurysm (AAA) is characterized by degeneration and progressive dilatation of the abdominal aorta wall. It has a prevalence of 4% to 7% in males over the age of 65, and 1% to 2% in females [1]. AAA rupture contributes to 150,000–200,000 deaths per year worldwide, making systemic screening of at-risk groups of great importance [2]. Tobacco use and age are the two strongest risk factors for AAA formation, whereas hypertension, dyslipidemia, and preexisting coronary artery disease (CAD) carry a relative risk of <1.5 [3]. Diabetes mellitus (DM) is negatively associated with AAA [4]. Currently, no medicine has been validated to halt the expansion of AAA, and surgical or endovascular repair are the only available options. Biomarkers with great sensitivity and specificity for AAA diagnosis and evaluation are not sufficient [5].

The molecular pathogenesis of AAA has been explored extensively. Particularly, an intense inflammatory response involving immune cell infiltration, MMP (matrix metalloproteinase) activation, cytokine production, and interaction between components of the vascular wall are considered crucial in AAA formation [3]. Autoimmunity as a trigger for AAA has received increasing interest in recent years [6,7]. B cell antibody production is hypothesized to respond to autoantigens resulting in the destruction of the vessel wall [7,8]. Researchers also found immunoglobulin G4 (IgG4)-positive plasma cell infiltration in the aortic wall of inflammatory aortic aneurysm [9]. However, the specific role of IgG in AAA needs further elucidation.

Glycosylation plays a pivotal role in modulating IgG effector function. IgG glycosylation is mostly N-glycosylation located at a highly conserved asparagine 297 (N297) glycosylation site in the “fragment crystallizable” (Fc) domain [10]. Alteration of glycan types could regulate the binding of IgG to complement proteins or Fc receptors, leading to changes in immunological function such as complement-dependent cytotoxicity (CDC), antibody-mediated cellular cytotoxicity (ADCC), and antibody-dependent cell-mediated phagocytosis (ADCP), etc., [10]. O-Glycosylation also takes place at the hinge region of IgG3 and may contribute to preventing IgG from proteolysis as well as facilitating Fab binding [11].

Among all techniques applied in glycosylation study, lectin microarray has the advantage of a simple and rapid procedure, high throughput, and high sensitivity without destruction of the native structure of glycans [12,13]. It is a powerful tool in glycan biomarker identification for diseases such as cancers and autoimmune diseases [14]. In this study, we firstly investigate the serum IgG glycosylation profile in patients with AAA and disease or healthy controls using a microarray containing 56 lectins. Significant differences were validated by lectin blot, and the clinical relevance of altered lectin binding was further explored.

## 2. Methods

### 2.1. Patients and Samples

From 2019 to 2021, 75 AAA patients from the Vascular Surgery ward of Peking Union Medical College Hospital (PUMCH) were recruited for the study. Disease controls, including 22 Takayasu disease (TA), 22 rheumatoid arthritis (RA), and 24 antiphospholipid syndrome (APS) patients matched by sex were included from the Department of Rheumatology. One hundred healthy controls from the health examination center matched by age and sex were also included. Serum samples were collected upon admission, allowed to clot at room temperature for 30 min, centrifuged for 5 min at 1000× *g*, and stored at −80 °C. No sample was exposed to more than one freeze–thaw cycle before analysis.

Patients’ demographic data, initial symptoms, as well as pre-operative laboratory results were collected. AAA patients were divided into subgroups according to concomitant diabetes, dyslipidemia, hypertension, and the degree of aortic wall expansion. Indication for repair included aneurysm diameters more than 5.5 cm for men or 5 cm for women and fast-growing (more than 10 mm/year) or symptomatic lesions [15]. The study was approved by the Ethics Committee of PUMCH, and all participants provided written informed consent.

### 2.2. Lectin Microarray Analysis

A total of 243 serum samples were analyzed using a commercial lectin microarray (BCBIO Biotech, Guangzhou, China) with 56 lectins, which had been proven for its reliability and used for biomarker discovery previously [16,17]. Briefly, lectin microarrays were taken out from −80 °C, warmed up at room temperature for half an hour, and incubated with a blocking buffer (3% BSA in PBS) at room temperature for 2 h afterward. After washing three times with PBST, 200 μL of 1:1000 diluted serum samples were added and incubated with the microarrays at 4 °C overnight. The microarrays were washed three times with PBST and then incubated with 5 mL of 1:1000 diluted Cy3-labeled goat anti-human IgG antibody (Jackson Immuno Research Labs, Pennsylvania, PA, USA) in the dark at room temperature for 1 h. Finally, after three PBST washes, microarrays were rinsed with D.I. water and dried. Microarrays were scanned with a GenePix 4000B Microarray Scanner (Molecular Devices, Sunnyvale, CA, USA).

For lectin array assays, the median foreground and background fluorescent intensity for each spot on the arrays were acquired using GenePix Pro 6.0 (Axon Instruments, CA, USA). We calculated the signal-to-noise ratio (S/N) (the medium intensity of the spot foreground relative to the background) of each lectin spot. To prevent bias of the lectin microarray from the inter-array, we normalized the S/N data in terms of quality control values between arrays.

### 2.3. Lectin Blot Verification

To validate the results of the differences in lectin microarray analysis, lectin blots were used to analyze serum samples collected from a smaller cohort, of which half were from the old study cohort and half were from a new set of patients. The following rules were used to identify lectins with significant binding activities: (a) fold change [group1(S/N)/group2(S/N)] ≥ 1.33 or <0.75, (b) *p*-value < 0.05.

Briefly, serum samples were first diluted using 1 × PBS, mixed with gel electrophoresis loading buffer (CW biotech, Beijing, China) to a final 1:100 ratio, and boiled for 10 min. Twenty microliters per sample was separated by 10% sodium dodecyl sulphate–polyacrylamide gel electrophoresis (SDS–PAGE) and electro-transferred onto polyvinylidene fluoride membranes (Millipore, Billerica, MA, USA). After washing two times, the membrane was incubated with 10× Carbo-Free Blocking Solution (1:10; Vector Laboratories Inc., Newark, CA, USA) at room temperature for 2 h. Then, the membranes were washed twice and incubated with 20 μg/mL of Cy3-labeled (1:1000; GE Healthcare, Chicago, IL, USA) lectins at 4 °C overnight in the dark. Finally, the washed and dried membranes were detected by a fluorescence signal system, Typhoon FLA 9500 (GE Healthcare, Chicago, IL, USA).

### 2.4. Statistical Analysis

R (v. 4.0.2) (R Core Team, Auckland, New Zealand) and GraphPad Prism 8.0.1 (Graph Pad Software, San Diego, CA, USA) were used for statistical analyses. The χ^2^ test or Fisher’s exact test were used for the comparison of categorical variables. For continuous variables, inter- or intra-group results were compared by Student’s t-test, Mann–Whitney U test, one-way analysis of variance (ANOVA) with Tukey’s HSD test, or Kruskal–Wallis test if appropriate. Pearson’s correlation coefficient was used to analyze the relationship between the lectin binding level and clinical indicators. A *p* value of less than 0.05 was considered statistically significant.

## 3. Results

### 3.1. Patient Characteristics

Table 1 summarizes the baseline information of all study subjects. Among AAA patients, 81.3% (61/75) were male, and the average age was 69.9 years old. Hypertension, dyslipidemia, and diabetes were presented in 80% (60/75), 56% (42/75), and 13.3% (10/75) of all patients, respectively. Fifty-six percent (42/75) of patients had prior smoking habits. The average hsCRP levels were 6.1 mg/L for AAA, 19.4 mg/L for TA, 12 mg/L for RA, 3.9 mg/L for APS, and 1.6 mg/L for the healthy controls. The average ESR levels were 17.2 mm/h for AAA, 21.5 mm/h for TA, 25.6 mm/h for RA, and 14.3 mm/h for APS patients. AAA patients had average blood HCY levels of 17.4 μmol/L and uric acid levels of 378.4 μmol/L. There were 48%, 74.7%, and 49.3% of AAA patients taking statins, antiplatelet therapy, and antihypertensive medication, respectively.

The detailed information on AAA patients is demonstrated in Table 2. The average aneurysm diameter was 52.5 mm among all patients, of which 56% had aneurysm diameters more than 5 cm. Symptoms were present in 28% of patients, and 44% of patients had concomitant iliac aneurysms. Patients with diabetes had an average HbA1c level of 6.9%.

### 3.2. Lectin Microarray Results

**Figure 1:**
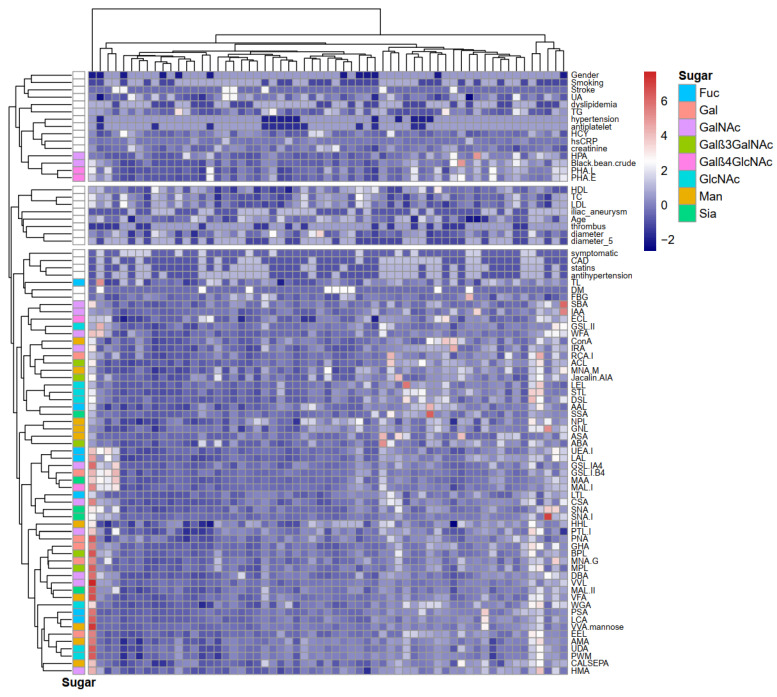
Heatmap of 56 lectin results from microarray analysis. Rows: samples; columns: lectins and clinical indicators. Preferred binding sugars for lectins were listed for each lectin. Color key indicates standardized fluorescent intensity for lectins, blue: lowest; red: highest. The heatmap was generated using R software (Version 4.0.2, https://cran.r-project.org/bin/windows/base/old/4.0.2/, accessed on 3 July 2021).

Results of all 56 lectins binding from the microarray underwent cluster analysis. As illustrated in Figure 1, lectin HPA (preferred GalNAc), black bean crude (preferred GalNAc), PHA-L (preferred Galβ4GlcNAc), and PHA-E (preferred Galβ4GlcNAc) were clustered together with creatinine, hsCRP, and other laboratory results or clinical indicators. TL (preferred GlcNAc) was clustered together with blood glucose levels.

**Figure 2:**
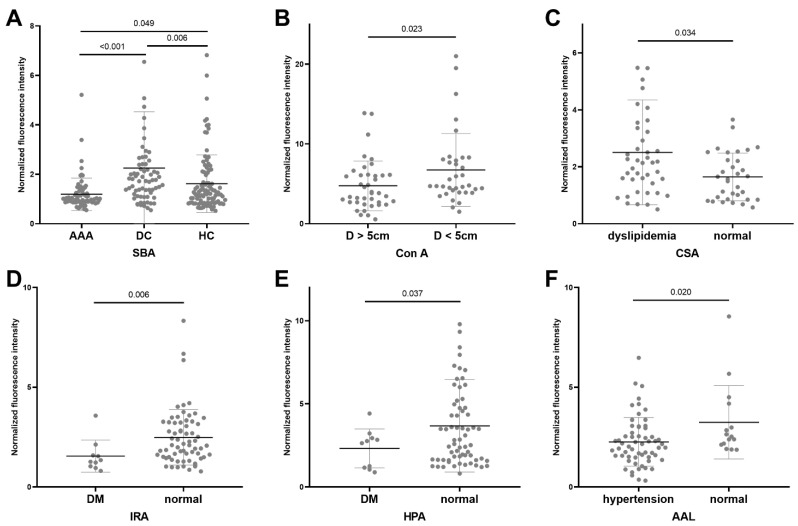
Significant lectin microarray results for AAA patients. The following rules were used to identify significant differences for intra-disease group analysis: (**A**) fold change [group1(S/N)/group2(S/N)] ≥ 1.33 or <0.75, (**B**) *p*-value < 0.05. (**A**) Microarray result for SBA in inter-group analysis. (**B**) Microarray result for ConA between patients with AAA of different diameters. (**C**) Microarray result for CSA between AAA patients with or without dyslipidemia. (**D**) Microarray result for IRA between AAA patients with or without diabetes. (**E**) Microarray result for HPA between AAA patients with or without dyslipidemia. (**F**) Microarray result for AAL between AAA patients with or without hypertension.

Lectins showing significant different binding activities are listed in Figure 2 and Table 3. Significantly lower binding level of SBA (preferred GalNAc) was observed for the AAA group compared with DCs (*p* < 0.001) and HCs (*p* = 0.049). A significantly lower binding level of ConA (preferred mannose) was observed for patients with aneurysm diameter >5 cm. Significantly higher binding of CSA (preferred GalNAc) was present for dyslipidemia patients, whereas a lower binding level of AAL (preferred fucose) was observed for hypertensive patients. Additionally, AAA patients with diabetes had lower binding levels of IRA (preferred GalNAc) and HPA (preferred GalNAc) compared with those not with DM.

### 3.3. Lectin Blot Analysis for AAA Patients

**Figure 3:**
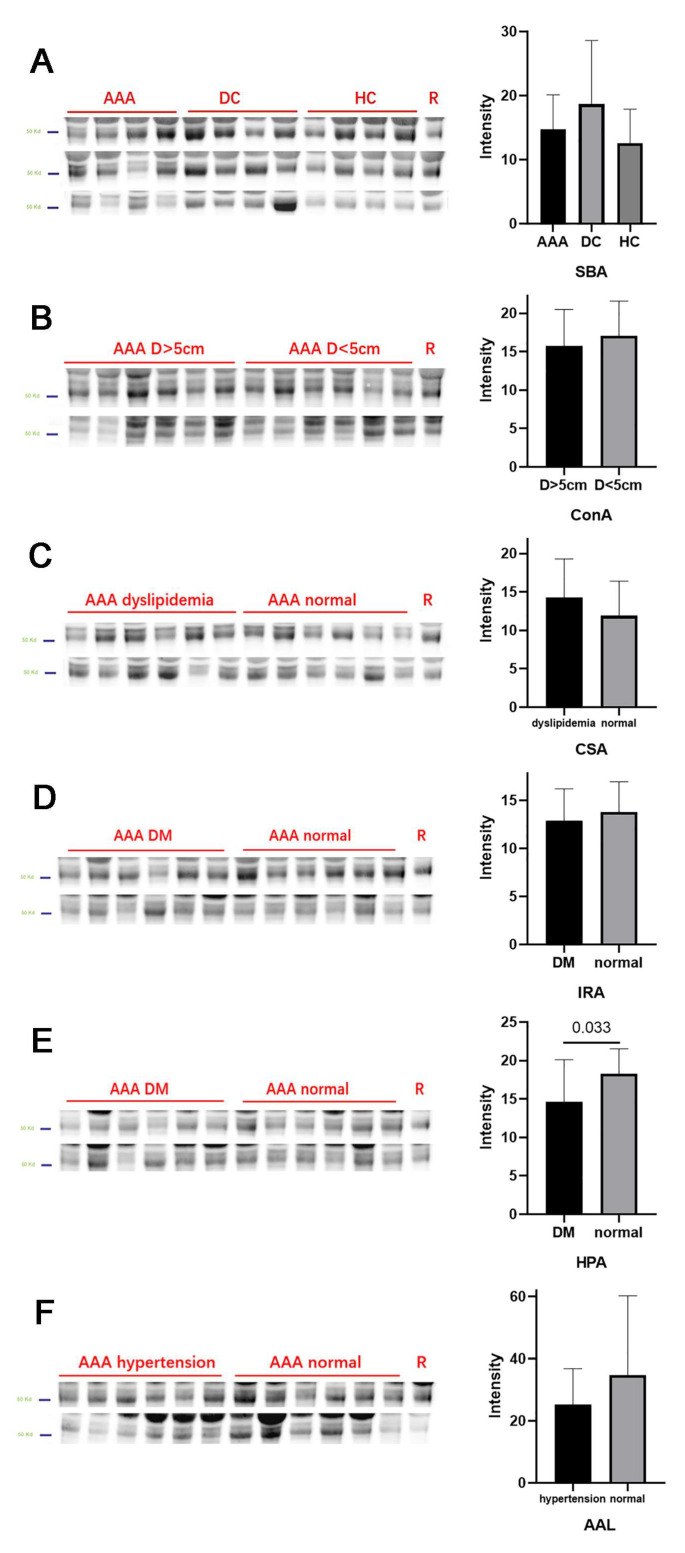
Lectin blot verification of microarray analysis. For each AAA subgroup, 6 old serum samples from the lectin microarray and 6 new samples were randomly selected for lectin blot. (**A**) Lectin blot result for SBA in inter-group analysis. (**B**) Lectin blot result for ConA between patients with AAA of different diameters. (**C**) Lectin blot result for CSA between AAA patients with or without dyslipidemia. (**D**) Lectin blot result for IRA between AAA patients with or without diabetes. (**E**) Lectin blot result for HPA between AAA patients with or without dyslipidemia. (**F**) Lectin blot result for AAL between AAA patients with or without hypertension.

Lectin blots were performed for selected significant lectins, with results shown in Figure 3. For each group/subgroup, six sera randomly selected from the microarray cohort together with six sera collected from a new cohort were included. The IgG bands are located at around 55 kd on SDS-PAGE. Similar tendencies of difference were observed in the lectin blots compared with the results from the microarray. Specifically, the binding level of HPA was significantly lower for diabetic patients than for non-diabetic patients.

### 3.4. Significant Clinical Relevance of Lectins

**Figure 4:**
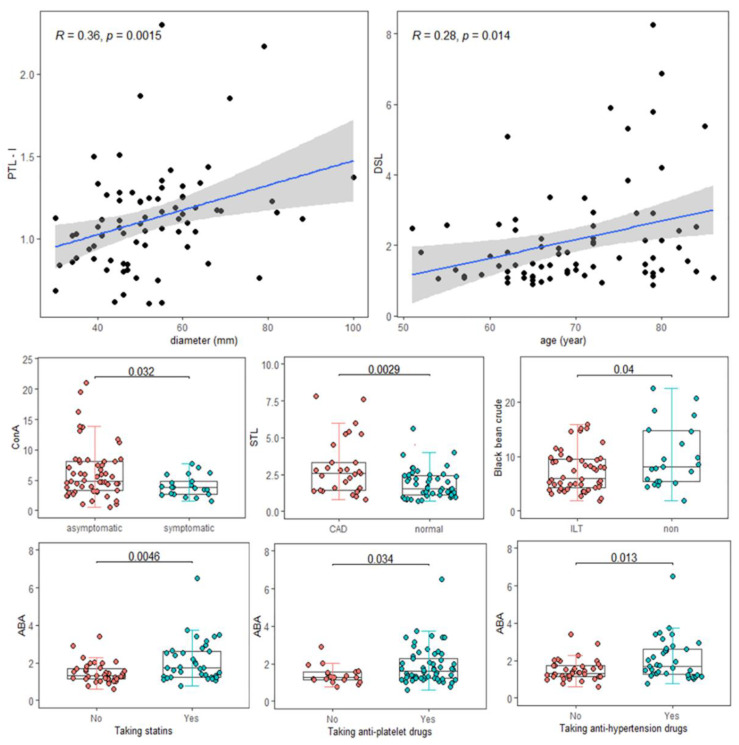
Clinical correlation of lectins with significant results from microarray. CAD, coronary heart disease. ILT, intraluminal thrombus. All AAA subjects were divided into two groups according to the presence of one factor (e.g., symptomatic) for intra-group analysis.

The clinical correlation of microarray results was calculated for each lectin, and significant results are shown in Figure 4. PTL-L (R = 0.36, *p* = 0.0015, preferred GalNAc) was positively associated with aneurysm diameters, whereas DSL (R = 0.28, *p* = 0.014, preferred (GlcNAc)2-4) was positively associated with patient age. Symptomatic patients had a lower binding level of ConA (*p* = 0.032), and patients with coronary heart disease had higher binding levels of STL (*p* = 0.0029, preferred GlcNAc). Patients with ILT bound less with black bean crude (*p* = 0.04, preferred GalNAc). For medication, patients taking statins (*p* = 0.0046), anti-platelet (*p* = 0.034) and anti-hypertension drugs (*p* = 0.013) had lower binding levels of ABA (preferred Galβ3GalNAc) compared with those not under medication.

## 4. Discussion

B cells and autoantibodies play an important role in the pathogenesis of AAA both through antigen-specific destruction of the arterial wall and activation of other immunoregulative cells [18]. Glycan variation occurs in more than 50% of human proteins and has a profound impact on the function of its binding components. Relatively well understood IgG glycosylations are highly valued as potential disease biomarkers and therapeutic targets [19]. Here, we explore the glycosylation pattern of serum IgG in AAA patients and controls with lectin microarray, seeking to identify new biomarkers for the diagnosis and evaluation of AAA.

For intergroup analysis, AAA had a significantly lower binding level of SBA (preferred glycan GalNAc) compared with disease and healthy controls. Lectin blots revealed similar results between AAA and DCs, yet the difference between AAA and HCs was not observed. Other lectins identified GalNAc were also found in AAA intragroup analysis. Patients diagnosed with diabetes had lower binding levels of IRA and HPA compared with those without. Patients with dyslipidemia had higher binding of CSA compared with those without. The result of HPA was confirmed by lectin blots. Moreover, we noticed that aneurysm diameter was positively associated with PTL-I, and patients with ILT had a lower binding level of black bean crude. GalNAc is a part of O-glycosylation, whose function has not been fully elucidated due to its resistance to proteolytic digestion and subsequential structural analysis [20,21]. Its alteration has been reported in some autoimmune diseases [17,22]. Stumer et al. found that GalNAc activity declined in RA patients receiving glucocorticoid therapy, proposing that it may be due to increased susceptibility of the IgG complex to be cleared [22]. In our study, GalNAc was positively associated with dyslipidemia, aneurysm diameter, and ILT, all of which indicate an increased risk or severity of AAA. Interestingly, GalNAc was negatively associated with diabetes, which is considered a protective factor for AAA. Our findings indicate that distinct O-glycosylation patterns are present in AAA patients, and might be associated with disease progression. Indeed, the binding level of lectin ABA (also preferred GalNAc) was higher in patients receiving three common medications for AAA (i.e., statins, antiplatelet, and antihypertensive agents). Measuring GalNAc-binding lectins may assist in the diagnosis and monitoring of AAA.

ConA, which preferably binds mannose glycans, was associated with aneurysm diameter <5 cm and symptomatic patients. Mannose-rich IgG N-glycan could initiate the MBL lectin complement cascade, resulting in subsequential immunological effects [19]. Symptoms such as pain or hypotension are a strong indication for AAA surgical intervention [23] and are also the main circumstances where aneurysms with smaller diameters required immediate repair. Our results suggest that a higher binding level of ConA might be helpful in identifying and risk stratification for these patients.

Age is one of the strongest risk factors for AAA. Our study revealed that bisecting GlcNAc-binding lectins was positively associated with patient age. Previous studies also revealed that increased levels of bisecting GlcNAc are present with higher age, whereas decreased levels were associated with longevity [24,25]. Additionally, consistent with results from some large cohorts [25,26], patients with concomitant CAD also showed increased binding of STL (preferred GlcNAc). GlcNAc could be a promising biomarker for reflecting systematic inflammatory states of cardiovascular diseases [27].

In addition, we also found that hypertensive patients had a lower binding level of AAL, which preferably recognizes the core fucose structure of Fc glycosylation. Lu et al. also observed a negative correlation between fucosylation and hypertension in both the Chinese and Croatian populations [28]. A lack of core fucose has been found to increase the ability of IgG to induce the ADCC effect [10]. Hypertension was hypothesized to be involved in T lymphocyte activation and vascular inflammation produced by the angiotensin II system [29]. Its relationship with IgG glycosylations and their impact on AAA formation still need clarification.

This study has some limitations. Due to a limited number of samples, the lectin blots did not verify all the results from the microarray. Although the serum level of IgG generally did not have much difference within AAA patients, it was not adjusted throughout the study in order for the convenience of biomarker discovery. The association between IgG glycosylation and other circulatory inflammatory markers still needs exploration. To further elucidate the structure of glycans and their roles in AAA pathogenesis, other techniques for glycan analysis including affinity chromatography and mass spectrometry would be combined in the future, and multi-regression analysis could be applied to consolidate the findings.

## 5. Conclusions

In conclusion, a decreased IgG binding level of SBA (recognizing glycan GalNAc) was observed in AAA patients compared with disease and healthy controls. Symptomatic patients with aneurysm <5 cm had a higher binding level of ConA (preferred mannose). IgG O-glycosylation (GalNAc) may play an important role in AAA pathogenesis and progression.

## Figures and Tables

**Table 1:** Clinical characteristics of patients.

No. (%) or Mean ± SD	AAA (*n* = 75)	TA (*n* = 22)	RA (*n* = 22)	APS (*n* = 24)	HC (*n* = 100)
Male, *n*%	61 (81.3)	18 (81.8)	18 (81.8)	20 (83.3)	81 (81)
Age (years)	69.9 ± 8.6	34.5 ± 8.1	61.0 ± 10.9	37.4 ± 12.2	66.5 ± 6.8
Hypertension, *n*%	60 (80)	10 (45.5)	59 (59)	4 (16.7)	59 (59)
Dyslipidemia, *n*%	42 (56)	0	3 (13.6)	0	48 (48)
Diabetes, *n*%	10 (13.3)	0	4 (18.2)	2 (8.3)	22 (22)
CAD, *n*%	29 (38.7)	0	2 (9.1)	3 (12.5)	NA
Stroke, *n*%	11 (14.7)	2 (9.1)	0	5 (20.8)	NA
Ever smoked, *n*%	42 (56)	2 (9.1)	10 (45.5)	11 (45.8)	NA
Total glycerol (mmol/L)	1.5 ± 0.8	0.7 ± 0.3	NA	5.3 ± 0.9	1.5 ± 0.8
LDL cholesterol (mmol/L)	2.6 ± 1.0	2.3 ± 0.9	NA	3.5 ± 0.6	3.0 ± 3.1
HDL cholesterol (mmol/L)	1.0 ± 0.2	1.2 ± 0.4	NA	NA	1.4 ± 0.6
Total cholesterol (mmol/L)	4.2 ± 1.2	4.1 ± 0.8	NA	2.1 ± 0.9	4.6 ± 1.1
HCY (μmol/L)	17.4 ± 8.2	13.8 ± 0.5	NA	NA	14.4 ± 3.2
hsCRP (mg/L)	6.1 ± 19.3	19.4 ± 38.2	12.0 ± 21.2	3.9 ± 7.2	1.6 ± 3.6
ESR (mm/h)	17.2 ± 15.4	21.5 ± 32.1	25.6 ± 24.3	14.3 ± 22.4	NA
Blood creatinine (μmol/L)	89.2 ± 33.3	79.8 ± 25.8	66.2 ± 14.1	NA	82.8 ± 21.4
Blood uric acid (μmol/L)	378.4 ± 80.9	307.9 ± 94.5	NA	NA	353.6 ± 84.8
FBG (mmol/L)	5.4 ± 1.3	4.9 ± 0.4	NA	NA	6.1 ± 1.7
HbA1c, %	6.1 ± 1.2	NA	NA	NA	5.9 ± 0.9
Medication, *n*%					
Statin	36 (48)	NA	NA	NA	NA
Antiplatelet	56 (74.7)	NA	NA	NA	NA
Antihypertensive	37 (49.3)	NA	NA	NA	NA

AAA, abdominal aortic aneurysm; TA, Takayasu aortitis; RA, rheumatoid arthritis; APS, antiphospholipid syndrome; HC, health control. CAD, coronary artery disease. LDL, low density lipoprotein. HDL, higher density lipoprotein. HCY, homocysteine. CRP, C-reactive protein. ESR, erythrocyte sedimentation rate. FBG, fasting blood glucose.

**Table 2:** Clinical characteristics for AAA patients.

No. (%) or Mean ± SD	All (*n* = 75)	Hypertension (*n* = 60)	Dyslipidemia (*n* = 42)	DM (*n* = 10)
Diameter, mm	52.5 ± 13.7	52.2 ± 13.3	50.4 ± 12.6	49.2 ± 10.1
Aneurysm diameter >5 cm, *n*%	42 (56)	35 (58.3)	19 (45.2)	5 (50)
ILT, *n*%	52 (69.3)	43 (71.7)	26 (61.9)	7 (70)
Symptom	21 (28)	15 (25)	14 (33.3)	4 (40)
Iliac aneurysm, *n*%	33 (44)	27 (45)	15 (35.7)	3 (30)
HbA1c, %	6.1 ± 1.2	6.1 ± 1.3	6.1 ± 0.8	6.9 ± 1.8

DM, diabetes mellitus. ILT, intraluminal thrombus.

**Table 3:** Sugar specificity for lectins with significant differences in inter- and intra-group analysis.

	Groups with Significant Change	Lectin Full Name	Monosaccharide Specificity
SBA	AAA vs. DC/HC, decrease	Soybean agglutinin	GalNAc
Con A	AAA > 5 cm vs. <5 cm, decrease	Concanavalin A	Mannose
CSA	AAA dyslipidemia vs. normal, increase	Cytisus sscoparius agglutinin	GalNAc
IRA	AAA DM vs. normal, decrease	Iris hybrid agglutinin	GalNAc
HPA	AAA DM vs. normal, decrease	Helix pomatia agglutinin	GalNAc
AAL	AAA hypertension vs. normal, decrease	Aleuria aurantia lectin	Fucose

All AAA subjects were divided into two groups according to the presence of one risk factor (e.g., dyslipidemia) for intra-group analysis.

## Data Availability

The datasets generated during and/or analyzed during the current study are available from the corresponding author on reasonable request.

## References

[1] Davis F.M., Daugherty A., Lu H.S. (2019). Updates of Recent Aortic Aneurysm Research. Arter. Thromb. Vasc. Biol..

[2] Golledge J. (2018). Abdominal aortic aneurysm: Update on pathogenesis and medical treatments. Nat. Rev. Cardiol..

[3] Quintana R.A., Taylor W.R. (2019). Cellular Mechanisms of Aortic Aneurysm Formation. Circ. Res..

[4] Shah A.D., Langenberg C., Rapsomaniki E., Denaxas S., Pujades-Rodriguez M., Gale C.P., Deanfield J., Smeeth L., Timmis A., Hemingway H. (2015). Type 2 diabetes and incidence of cardiovascular diseases: A cohort study in 1·9 million people. Lancet Diabetes Endocrinol..

[5] Stather P.W., Sidloff D.A., Dattani N., Gokani V.J., Choke E., Sayers R.D., Bown M. (2014). Meta-analysis and meta-regression analysis of biomarkers for abdominal aortic aneurysm. Br. J. Surg..

[6] Tilson M.D. (2016). Decline of the atherogenic theory of the etiology of the abdominal aortic aneurysm and rise of the autoimmune hypothesis. J. Vasc. Surg..

[7] Furusho A., Aoki H., Ohno-Urabe S., Nishihara M., Hirakata S., Nishida N., Ito S., Hayashi M., Imaizumi T., Hiromatsu S. (2018). Involvement of B Cells, Immunoglobulins, and Syk in the Pathogenesis of Abdominal Aortic Aneurysm. J. Am. Heart Assoc..

[8] Ando T., Iizuka N., Sato T., Chikada M., Kurokawa M.S., Arito M., Okamoto K., Suematsu N., Makuuchi H., Kato T. (2013). Autoantigenicity of carbonic anhydrase 1 in patients with abdominal aortic aneurysm, revealed by proteomic surveillance. Hum. Immunol..

[9] Li H., Bai S., Ao Q., Wang X., Tian X., Li X., Tong H., Hou W., Fan J. (2018). Modulation of Immune-Inflammatory Responses in Abdominal Aortic Aneurysm: Emerging Molecular Targets. J. Immunol. Res..

[10] Quast I., Peschke B., Lünemann J.D. (2017). Regulation of antibody effector functions through IgG Fc N-glycosylation. Cell. Mol. Life Sci..

[11] Plomp R., Dekkers G., Rombouts Y., Visser R., Koeleman C.A., Kammeijer G.S., Jansen B.C., Rispens T., Hensbergen P.J., Vidarsson G. (2015). Hinge-Region O-Glycosylation of Human Immunoglobulin G3 (IgG3). Mol. Cell. Proteom..

[12] Hirabayashi J., Yamada M., Kuno A., Tateno H. (2013). Lectin microarrays: Concept, principle and applications. Chem. Soc. Rev..

[13] Hirabayashi J., Kuno A., Tateno H. (2014). Development and Applications of the Lectin Microarray. SialoGlyco Chem. Biol. II.

[14] Dang K., Zhang W., Jiang S., Lin X., Qian A. (2020). Application of Lectin Microarrays for Biomarker Discovery. ChemistryOpen.

[15] Erbel R., Aboyans V., Boileaul C., Bossone E., Bartolomeo R.D., Eggebrecht H., Evangelista A., Falk V., Frank H., Gaemperli O. (2014). ESC Guidelines on the diagnosis and treatment of aortic diseases: Document Covering Acute and Chronic Aortic Diseases of the Thoracic and Abdominal Aorta of the Adult. The Task Force for the Diagnosis and Treatment of Aortic Diseases of the European Society of Cardiology (ESC). Eur. Heart J..

[16] Sun Y., Cheng L., Gu Y., Xin A., Wu B., Zhou S., Guo S., Liu Y., Diao H., Shi H. (2016). A Human Lectin Microarray for Sperm Surface Glycosylation Analysis. Mol. Cell. Proteom..

[17] Zeng X., Li S., Tang S., Li X., Zhang G., Li M., Zeng X., Hu C. (2021). Changes of Serum IgG Glycosylation Patterns in Primary Biliary Cholangitis Patients. Front. Immunol..

[18] Zhang L., Wang Y. (2015). B lymphocytes in abdominal aortic aneurysms. Atherosclerosis.

[19] Russell A., Adua E., Ugrina I., Laws S., Wang W. (2018). Unravelling Immunoglobulin G Fc N-Glycosylation: A Dynamic Marker Potentiating Predictive, Preventive and Personalised Medicine. Int. J. Mol. Sci..

[20] De Haan N., Falck D., Wuhrer M. (2019). Monitoring of immunoglobulin N- and O-glycosylation in health and disease. Glycobiology.

[21] Chu T.H., Patz E.F., Ackerman M.E. (2021). Coming together at the hinges: Therapeutic prospects of IgG3. mAbs.

[22] Stümer J., Biermann M.H.C., Knopf J., Magorivska I., Kastbom A., Svärd A., Janko C., Bilyy R., Schett G., Sjöwall C. (2017). Altered glycan accessibility on native immunoglobulin G complexes in early rheumatoid arthritis and its changes during therapy. Clin. Exp. Immunol..

[23] Rokosh R.S., Wu W.W., Schermerhorn M., Chaikof E.L. (2021). Society for Vascular Surgery implementation of clinical practice guidelines for patients with an abdominal aortic aneurysm: Postoperative surveillance after abdominal aortic aneurysm repair. J. Vasc. Surg..

[24] Ruhaak L.R., Uh H.-W., Beekman M., Koeleman C.A.M., Hokke C.H., Westendorp R.G.J., Wuhrer M., Houwing-Duistermaat J.J., Slagboom P.E., Deelder A.M. (2010). Decreased Levels of Bisecting GlcNAc Glycoforms of IgG Are Associated with Human Longevity. PLoS ONE.

[25] Menni C., Gudelj I., Macdonald-Dunlop E., Mangino M., Zierer J., Bešić E., Joshi P.K., Trbojević-Akmačić I., Chowienczyk P.J., Spector T.D. (2018). Glycosylation Profile of Immunoglobulin G Is Cross-Sectionally Associated with Cardiovascular Disease Risk Score and Subclinical Atherosclerosis in Two Independent Cohorts. Circ. Res..

[26] Tibuakuu M., Fashanu O.E., Zhao D., Otvos J.D., Brown T.T., Haberlen S.A., Guallar E., Budoff M.J., Palella F.J., Martinson J.J. (2019). GlycA, a novel inflammatory marker, is associated with subclinical coronary disease. AIDS.

[27] Dashti H., Porras M.A.P., Mora S. (2021). Glycosylation and Cardiovascular Diseases. Adv. Exp. Med. Biol..

[28] Lu J.-P., Knežević A., Wang Y.-X., Rudan I., Campbell H., Zou Z.-K., Lan J., Lai Q.-X., Wu J.-J., He Y. (2011). Screening Novel Biomarkers for Metabolic Syndrome by Profiling Human Plasma N-Glycans in Chinese Han and Croatian Populations. J. Proteome Res..

[29] Marvar P.J., Thabet S.R., Guzik T.J., Lob H.E., McCann L.A., Weyand C., Gordon F.J., Harrison D.G. (2010). Central and Peripheral Mechanisms of T-Lymphocyte Activation and Vascular Inflammation Produced by Angiotensin II–Induced Hypertension. Circ. Res..

